# Editorial: Eating Disorders From Binge to Anorexia: Basic and Clinical Approaches for a Translational Research

**DOI:** 10.3389/fnut.2021.678451

**Published:** 2021-05-14

**Authors:** Virginie Tolle, Odile Viltart

**Affiliations:** ^1^Université de Paris, UMR-S 1266 INSERM, Institute of Psychiatry and Neuroscience of Paris (IPNP), Paris, France; ^2^Université de Lille, Faculté des Sciences et Technologies, Villeneuve d'Ascq, France

**Keywords:** eating behavior, hypothalamus, reward, goal-directed behavior, autonomic nervous system, animal models, epigenetic

Eating disorders (ED) cover a variety of psychiatric illnesses (anorexia nervosa/AN, bulimia nervosa, and binge eating) in which individuals express abnormal eating behaviors, often resulting in either insufficient or excessive food intake. The challenge is to understand how the eating behavior turns aberrant to the point that it becomes life threatening. In mammals, food intake involves both peripheral and central effectors directly linked to energy homeostasis regulation. It integrates neuronal circuitries that regulate stress, emotions, reward, biological rhythms, learning, and individual experience with food. In humans, it includes cognitive processes and socio-cultural and genetic factors. Thus, eating depends on the functioning of a hard-wired homeostatic circuitry–that is almost entirely identical between mammals because of evolutionary selection–together with a more flexible non-homeostatic hedonic circuitry, the functions of which vary according to individuals' experiences and/or epigenetic variations. Translational research, by bridging the gap between basic research and medical practice, allows for assessing the complex crosstalk between the central nervous system and peripheral organs, to better decipher the mechanisms leading to ED.

The aim of this Research Topic was to gather pioneering articles from experts in the field to better understand the multifactorial facets of ED. It is a compilation of nine original research articles, review papers, or perspectives written by 55 authors, covering pre-clinical, translational, and clinical aspects.

The phenomenology of ED relates to altered functioning of various systems, including the autonomic nervous system (ANS). As the stress response is an integral part of the ANS, the original and methodological paper of Simões-Capela et al. designed an experiment allowing the experimenter to capture ANS tonic variations and phasic activations in response to stressors in patients suffering from various ED. These devices are easy to use and can be adapted to different clinical conditions. They will help in defining a dimensional biologically-oriented nosology and establish a multiparametric model of the ED spectrum.

Two original research articles explored inter-individual variability in the susceptibility to developing ED. De Francesco et al. showed that inbred C57BL/6 mice display a large degree of inter-individual variability in high-fat consumption. Higher consumption of a high-fat diet was associated with a higher activation of mesencephalic dopamine neurons, suggesting a differential response to the rewarding properties of the high-fat diet in high vs. low consumers. Brenachot et al. showed that inter-individual vulnerability to developing maladaptive eating and obesity induced by the diet also depends on homeostatic circuits involving the polysialic acid-neural cell adhesion molecule, a key regulator of brain plasticity. The cross-talk between peripheral signals and hypothalamic circuits has long been shown to control hunger as well as a wide range of behaviors such as motivation, locomotor activity, negative reinforcement, and anxiety, as highlighted in the review by Méquinion et al. They underlined the potential role of hypothalamic AgRP neurons and ghrelin signaling in both the metabolic and behavioral adaptations to undernutrition in AN, a disorder with both psychiatric and metabolic etiology.

Amongst the brain modifications induced by the chronic self-induced food restriction observed in AN patients, the meta-analysis of Zhang et al. aimed to identify the most consistent white matter abnormalities between AN patients and healthy controls using ≪ seed-based d mapping ≫, a statistical technique for meta-analyzing studies that uses neuroimaging techniques to investigate the changes in brain activity or structure. This first quantitative meta-analysis revealed, in AN, microstructural differences in the interhemispheric connections and limbic association fibers.

Modeling AN in laboratory animals is a challenge since this psychiatric disorder is characterized by voluntary food restriction. Most animal models developed to date attempt to mimic core features of human AN. The systematic review of Schalla and Stengel on the Activity-Based-Anorexia (ABA) rodent model recapitulates a wide range of cellular, molecular, and behavioral changes (brain, hormonal, and immune alterations) that may underline AN. The review also highlights potential future developments such as the use of pharmacological interventions or chronic adaptations to the ABA model. Besides this “environmental” model of AN, Maltais et al. (1) described a genetic model of AN, the *anx/anx* mouse, which displays a spontaneous mutation on the chromosome 2 between the markers D2Mit133 and Jojo5 (2). The review by Nilsson depicted how this model can be considered as a valuable natural model to explore the (neuro)biology of AN and better understand their abnormal response to negative energy balance. Indeed, these mice share several characteristics with AN patients (emaciation, starvation, or pre-mature death). They also display changes in hypothalamic neuro-peptidergic and -transmitter systems that regulate food intake and metabolism, accompanied by hypothalamic inflammation and degeneration and by alterations in glucose homeostasis and pancreatic function.

However, AN is a disorder with a complex and multifactorial etiology, making it difficult to model in living organisms. Thanks to *in vitro* genomic and epigenomic analyses, identification of multiple genetic, epigenetic, and cellular bases of AN has been possible. Maussion et al. suggest to use the power of induced pluripotent stem cells combined with CRISPR–Cas9 technology as a tool to investigate new molecular and cellular mechanisms for this disorder. The authors present cellular, molecular, and whole organism models that will help to improve our current understanding of the pathophysiology of ED and provide new therapeutic strategies to address specific endophenotypes.

Finally, the review by Södersten et al. discussed current opinions about the etiology of AN and presented a successful approach to treat this disorder and achieve a low level of relapse. The review suggests that the standard perspective to consider AN, i.e., as a neurochemically and genetically mediated mental illness, needs to be adjusted because the clinical treatment classically used (cognitive therapy) is at a standstill. The authors discussed the validity of the evolutionary perspective that includes, for example, physical activity as an adaptation for food seeking. Based on a mathematical model, they explained their successful experience in treating AN using a method in which patients restore their eating behavior by practicing eating. The control of eating behavior is therefore outsourced to a machine that provides feedback on how to eat.

## Author Contributions

All authors listed have made a substantial, direct and intellectual contribution to the work, and approved it for publication.

## Conflict of Interest

The authors declare that the research was conducted in the absence of any commercial or financial relationships that could be construed as a potential conflict of interest.
